# *HLA-DR* genetic polymorphisms and hepatitis B virus mutations affect the risk of hepatocellular carcinoma in Han Chinese population

**DOI:** 10.1186/s12985-023-02253-2

**Published:** 2023-11-30

**Authors:** Yubao Zhao, Kun Chen, Hui Yang, Feng Zhang, Lu Ding, Yan Liu, Le Zhang, Yuchen Zhang, Huiliang Wang, Yang Deng

**Affiliations:** 1 Department of Infectious Diseases, Second Affiliated Hospital of Shandong First Medical University, 706 Taishan Street, Tai’an, Shandong Province China; 2 School of Public Health, Shandong First Medical University & Shandong Academy of Medical Sciences, 6699 Qingdao Road, Jinan, Shandong Province China; 3 Department of Gastrointestinal Surgery, Tai’an Central Hospital, 29 Longtan Road, Tai’an, Shandong Province China; 4 Department of Public Health, Jinan Central Hospital, 105 Jiefang Road, Jinan, Shandong Province China

**Keywords:** Hepatitis B virus, Hepatocellular carcinoma, Human leukocyte antigen-DR, Mutation, Single nucleotide polymorphism

## Abstract

**Background:**

Human leucocyte antigen (*HLA*)*-DR* plays a crucial role in the immune response against hepatitis B virus (HBV). We aimed to investigate the associations of *HLA-DR* single nucleotide polymorphisms (SNPs) with the generation of hepatocellular carcinoma (HCC)-related HBV mutations. The effects of *HLA-DR* SNPs and their interactions with HBV mutations on HCC risks were also determined.

**Methods:**

Five *HLA-DR* SNPs (rs3135363, rs9268644, rs35445101, rs24755213, and rs984778) were genotyped in 792 healthy controls, 586 chronic hepatitis B (CHB) patients, 536 liver cirrhosis (LC) patients, and 1500 HCC patients using quantitative PCR. Sanger sequencing was used to identify the HBV mutations. Logistic regression model was performed to evaluate the association of *HLA-DR* SNPs with HCC risk and the frequencies of HCC-related HBV mutations.

**Results:**

The variant genotypes at rs3135363, rs9268644, rs35445101, rs24755213, and rs984778 were associated with decreased HCC risks. In genotype C HBV-infected subjects, variant genotypes of these SNPs were associated with decreased frequencies of HCC-related HBV mutations such as C1653T, T1674C/G, G1719T, T1753A/C, A1762T/G1764A, A1846T, G1896A, G1899A, and preS deletion. AG genotype at rs3135363, CA genotype at rs9268644, and AG genotype at rs24755213 reduced the generation of T1753A/C and G1896A in genotype B HBV-infected subjects, respectively. In addition, the interactions of rs3135363, rs9268644, rs24755213 with C1653T, T1753A/C, A1846T, and G1896A decreased the risks of HCC.

**Conclusions:**

*HLA-DR* genetic polymorphisms might predispose the host to immunoselection of HCC-related HBV mutations and affect the HCC risks possibly through interacting with HBV mutations.

**Supplementary Information:**

The online version contains supplementary material available at 10.1186/s12985-023-02253-2.

## Introduction

According to the global cancer estimate for 2020, liver cancer ranks as the sixth most commonly diagnosed cancer as well as the third most common cause of death due to cancer [1, 2]. Hepatocellular carcinoma (HCC) is the principal histologic type of liver cancer, accounting for about 90% of all cases [3]. Chronic infection with hepatitis B virus (HBV) contributes more than 50% of global HCC cases [4]. HBV is identified as one of the Group 1 human carcinogens for HCC by World Health Organization (WHO) [5, 6]. HCC also remains the second leading cause of premature death from cancer [2]. Aetiology-specific prophylaxis is considered to be one of the most cost-effective strategies to decrease HCC-caused immature deaths [7]. Thus, it poses a great challenge to identify the HBV-infected individuals who are more susceptible to develop HCC.

The natural history of chronic HBV infection includes immune tolerance (IT), immune clearance, hepatitis B e antigen (HBeAg)-negative inactive/quiescent carrier, and HBeAg-negative hepatitis phases [8]. In the initial of IT phase, HBV is usually wild-type when HBeAg is positive as well as viral load of HBV DNA is high and immune response is weak. HBV mutations gradually occur in the progression to the later phases, especially during HBeAg seroconversion [9, 10]. Some HBV mutations, predominantly those in the enhancer II/basal core promoter/precore (EnhII/BCP/PC) and preS regions of HBV genome, are associated with the increased risk of HCC and poor prognosis of HCC patients after hepatectomy [11–14]. These HBV mutations, for example, C1653T, T1753A/C, A1762T/G1764A, and preS mutations, are termed HCC-related HBV mutations [15–17]. HCC-related HBV mutations may appear several years before the diagnosis of HCC and gradually accumulate during the progression to advanced stages of HBV-related liver diseases [9, 15, 16]. For example, a well-known HCC-related HBV mutation A1762T/G1764A, which is prevalent in more than 70% HBV-infected HCC patients, occurs approximately ten years before the diagnosis of HCC [16, 18]. Thus, the occurrence of HCC-related HBV mutations can serve as potential biomarkers for predicting the outcomes of chronic HBV infection, particularly with regard to patients who will develop HCC.

Human leukocyte antigen (HLA) class II molecules contain HLA-DR, DP and DQ that play crucial roles in presenting antigenic peptides on the cell surface for recognition by T cell receptors to activate CD4^+^ T cell-mediated immunity, as well as regulating of overall immune responses [19, 20]. Genetic predisposition of HLA class II antigens probably acts on the immune imbalance upon HBV infection, leading to chronic liver inflammation [21]. Previous studies have revealed that single nucleotide polymorphisms (SNPs) in HLA class II alleles are associated with the susceptibility to chronic HBV infection and hepatocarcinogenesis [7, 10, 21–25]. For example, the variant genotypes at rs3077, rs3135021, and rs9277535 located in *HLA-DPA1* and *HLA-DPB1* regions were inversely associated with HBV persistence [10]. The variant genotypes of two SNPs in *HLA-DQ* region (rs2856718 and rs9275319) were significantly associated with a decreased HCC risk [21]. However, the associations of *HLA-DR* SNPs with HCC risk have not been fully clarified, except for our previous study reporting a regulatory effect of rs477515 (located in the enhancer of *HLA-DRB1*) on genetic susceptibility to HCC.^7^

In this study, we conducted a case-control study with a large sample size to investigate the associations of *HLA-DR* SNPs with the generation of HCC-related HBV mutations, and evaluate the effects of *HLA-DR* SNPs and their interactions with HBV mutations on the risks of HCC. This study may be useful in identifying HBV-infected subjects who are more susceptible to develop HCC and need specific prophylaxis for HCC.

## Methods

### Study subjects

In this study, we initially enrolled healthy controls, patients with chronic hepatitis B (CHB), patients with liver cirrhosis (LC), and patients with HCC from the Second Affiliated Hospital of Shandong First Medical University, Tai’an Central Hospital, and Jinan Central Hospital between September 2019 and April 2023. Healthy controls were recruited from healthy individuals who received routine physical examinations, and they were free of HBV and/or hepatitis C virus infection and had no history of liver diseases. The CHB patients were diagnosed according to the following criteria: (i) seropositive for HBsAg for more than 6 months; (ii) high level of serum HBV DNA (Hepatitis B e antigen [HbeAg]-positive subjects: > 20,000 IU/mL, HBeAg-negative subjects: > 2,000 IU/mL); (iii) persistent or intermittent elevation in alanine and/or aspartate aminotransferase (ALT/AST) levels; (iv) liver biopsy showing chronic hepatitis. LC patients were diagnosed by histologic analysis or ultrasonography test with complications in portal hypertension. The diagnostic criteria of HCC patients were included: (i) diagnosed on the basis of cytologic or pathologic analysis; (ii) positive image on computerized tomography or magnetic resonance imaging; (iii) an elevated α-fetoprotein ≥ 400 ng/mL. All study subjects were ethnic Han Chinese and gave written informed consents. Five milliliters of peripheral blood were collected from each subject, and the sera and genomic DNA were isolated and stored at -80℃ within 4 h of collection for further tests. The study was conducted in accordance with the 1975 Declaration of Helsinki and was approved by the ethics committee of Shandong First Medical University & Shandong Academy of Medical Sciences (Ethics Approval No. 2022S6010, 8 March 2022). All information, including clinical and virological data, was recorded in an anonymized database.

### Serological viral marker testing, clinical characteristics, HBV genotyping and mutation analysis

Serological viral markers including HBsAg, HBeAg, anti-HBs, anti-HBe, and anti-HBc were detected with enzyme-linked immunosorbent assay kits (Kehua, Shanghai, China) according to the manufacturer’s protocols. Clinical characteristics including ALT, AST, total bilirubin (TBiL), direct bilirubin (DBiL), alkaline phosphatase (ALP), glutamyl transpeptidase (GGT), albumin (ALB), and platelet (PLT) were examined in the hospitals at the enrollment. HBV genotyping and HBV genome DNA amplification were determined by multiplex nested polymerase chain reaction (PCR) as described in the previous study [7, 11, 12]. Detailed information regarding the primers, PCR reaction system, and conditions were provided in the Supplementary materials. The nucleotide which has the highest frequency in the sequences of HBV genome DNA from HBeAg-positive asymptomatic HBsAg carrier was identified as a wild-type nucleotide because HBeAg-positive HBV was considered as a wild-type strain [26, 27]. A nucleotide substitution and deletion at each site was termed as HBV mutation [10]. Sequence alignment and mutation analysis of HBV genome were performed by using MEGA 4.0 software.

### Selection of HLA-DR SNPs and genotyping

Five representative *HLA-DR* SNPs with a minor allele frequency of > 5% in Han Chinese according to the 1000 Genomes Project (https://www.internationalgenome.org/) were selected. rs3135363 (-18kb, *HLA-DRA*, 5’ upstream, A > G) and rs984778 (-7.5kb, *HLA-DRA*, 5’ upstream, T > C) were selected because they were associated with immune responsiveness to HBV vaccinations [28, 29]. rs9268644 (+ 380 bp, *HLA-DRA*, intron 1, C > A) was selected because the minor A allele (AA + AC) of rs9268644 had a protective effect on hepatitis in the Korean population [30]. rs35445101 (+ 10.7kb, *HLA-DRB1*, exon 6, A > G) was selected because it was associated with TP53 expression status in HBV-infected HCC [31]. We selected rs24755213 as a candidate SNP because it was a functional SNP in the *HLA-DRB1* haplotype block, which was determined by using Haploview 4.2 software. Fluorescent probe real-time quantitative PCR was performed for SNP genotyping in the LightCycler^TM^480 (Roche, Basel, Switzerland). The sequences of TaqMan probes and primers for five SNPs, PCR reaction system, and condition were listed in Supplementary Table S1.

### Statistical analyses

Hardy-Weinberg equilibrium (HWE) of each SNP was examined online (http://ihg.gsf.de/ihg/snps.htm) in the healthy controls. Differences in continuous variables among different groups were tested by one-way analysis of variance analysis and corrected using the Bonferroni correction for multiple comparisons. Chi-square test was conducted to analyze the difference in categorical variables. Because the sequence of HBV wild-type differs considerably from HBV genotypes B and C [15], we performed the HBV mutation analysis in each stratum stratified by HBV genotype. An unconditional logistic regression model was performed to evaluate the association of *HLA-DR* SNPs with HCC risk and the frequencies of HCC-related HBV mutations, expressed by odds ratios (ORs) and corresponding 95% confidence intervals (CIs). The multiplicative interactions of HBV mutations with *HLA-DR* SNPs on the HCC risk were determined by multivariate logistic regression. All statistical tests were two-sided and performed using the SPSS24.0 (SPSS, Chicago, IL), with a *P* value of < 0.05 representing statistically significant.

## Results

### Characteristics of study subjects

A total of 3,414 participants including 792 healthy controls, 586 CHB patients, 536 LC patients, and 1500 HCC patients were included in the case-control study. The demographic and clinical characteristics of these participants were shown in Table 1. HCC patients were older than HBV-infected subjects without HCC (*P* < 0.001), while HCC patients were younger than healthy controls (*P* = 0.001). Male and genotype C were more frequent in HCC patients than subjects without HCC, whereas the proportion of HBeAg positivity was lower in HCC patients than subjects without HCC. Higher levels of HBV DNA, ALT, AST, ALB and PLT were observed in HBV-infected subjects without HCC than HCC patients. TBiL, DBiL, ALP, and GGT levels in HBV-infected subjects with HCC were higher than those without HCC.

**Table 1:** The demographic and clinical characteristics of study subjects

Characteristic	Healthy controls (n = 792)	CHB (n = 586)	LC (n = 536)	HCC (n = 1500)	*P* value
Male (%)	488 (61.62)	416 (70.99)	391 (72.95)	1257 (83.80)	0.001^a^, < 0.001^b,c^
Age, years (mean ± SD)	55.57 ± 13.06	43.99 ± 14.65	50.46 ± 10.84	53.02 ± 11.30	0.001^a^, < 0.001^b,c^
HBeAg (%)					
Positive	ND	259 (44.20)	202 (37.69)	450 (30.00)	< 0.001^b^
Negative	ND	327 (55.80)	334 (62.31)	1050 (70.00)	
Genotype (%)	ND				
B	ND	103 (23.62)	86 (19.91)	185 (16.53)	0.003^b^
C	ND	333 (76.38)	346 (80.09)	934 (83.47)	
HBV DNA load, log_10_ IU/mL (mean ± SD)	ND	3.68 ± 0.91	3.45 ± 0.60	3.10 ± 0.44	< 0.001^b^
ALT, U/L (mean ± SD)	20.47 ± 17.95	222.87 ± 497.96	124.14 ± 195.77	76.09 ± 171.97	< 0.001^a,b,c^
AST, U/L (mean ± SD)	21.89 ± 13.44	133.35 ± 206.01	115.02 ± 133.44	83.81 ± 184.01	< 0.001^a,b,c^
TBiL, μmol/L (mean ± SD)	12.91 ± 4.07	23.10 ± 35.52	54.97 ± 82.78	51.45 ± 89.83	< 0.001^a,b,c^
DBiL, μmol/L (mean ± SD)	4.64 ± 7.28	9.45 ± 14.07	27.68 ± 50.89	25.78 ± 54.94	< 0.001^a,b,c^
ALP, U/L (mean ± SD)	69.75 ± 25.41	109.08 ± 41.88	131.57 ± 61.38	141.49 ± 103.98	< 0.001^a,c^, 0.035^b^
GGT, U/L (mean ± SD)	27.32 ± 24.65	87.86 ± 106.36	89.49 ± 131.91	157.80 ± 184.02	< 0.001^a,b,c^
ALB, g/L (mean ± SD)	44.25 ± 5.06	42.30 ± 4.75	34.28 ± 7.31	36.84 ± 7.19	< 0.001^a,b,c^
PLT, 10^9^/L (mean ± SD)	205.10 ± 41.22	152.78 ± 43.47	86.43 ± 24.59	100.34 ± 28.44	< 0.001^a,b,c^

^a^ HBV-infected subjects with HCC versus. Healthy controls

^b^ HBV-infected subjects with HCC versus. HBV-infected subjects without HCC.

^c^ HBV-infected subjects without HCC versus. Healthy controls

*ALB* albumin, *ALP* alkaline phosphatase, *ALT* alanine aminotransferase, *AST* aspartate transaminase, *CHB* chronic hepatitis B, *DBiL* direct bilirubin, *GGT* glutamyl transpeptidase, *HBeAg* hepatitis B e antigen, *HBV* hepatitis B virus, *HCC* hepatocellular carcinoma, *LC* liver cirrhosis, *ND* no data, *PLT* platelet, *SD* Standard deviation, *TBiL* total bilirubin

Statistically significant levels were corrected using the Bonferroni correction for multiple comparisons (*P* = 0.017)

### Associations of ***HLA-DR*** genetic polymorphisms with HCC risk

The successful detection rates of rs3135363, rs9268644, rs35445101, rs24755213, and rs984778 were 98.87%, 98.74%, 99.18%, 98.86%, and 99.08%, respectively. The genotype frequencies of rs3135363, rs9268644, rs24755213, and rs984778 were conformed to HWE in healthy controls, except rs35445101 (Supplementary Table S2). We amplified a DNA fragment covering rs35445101 and sequenced in 50 randomly selected healthy controls. The genotyping results of Sanger sequencing were 100% concordant with quantitative PCR results. The genotypes distributions of the five *HLA-DR* SNPs and their association with HCC risk were presented in Table 2. Compared to healthy controls, the variant genotypes at rs3135363, rs9268644, rs35445101, rs24755213, and rs984778 were associated with decreased HCC risks. Compared to HBV-infected subjects without HCC, the GG genotype at rs3135363, variant genotypes including CA genotype, AA genotype, and A allele at rs9268644, GG genotype and G allele at rs35445101 were associated with reduced HCC risks.

**Table 2:** Associations of *HLA-DR* genetic polymorphisms with HCC risk

SNPs	Genotype	Healthy controls (%)	CHB plus LC (%)	HCC (%)	HCC versus. Healthy controls			HCC versus. HBV-infected subjects without HCC	
					AOR^a^ (95% CI)	*P* value		AOR^b^ (95% CI)	*P* value
rs3135363	AA	465 (58.71)	712 (63.46)	1006 (67.07)	Reference			Reference	
	AG	276 (34.85)	331 (29.50)	438 (29.20)	**0.74 (0.61–0.89)**	**0.001**		0.93 (0.79–1.11)	0.434
	GG	51 (6.44)	79 (7.04)	56 (3.73)	**0.52 (0.35–0.77**	**0.001**		**0.51 (0.36–0.73)**	**1.97 × 10** ^**− 4**^
	AG + GG	327 (41.29)	410 (36.54)	494 (32.93)	**0.70 (0.55–0.90)**	**9.59 × 10** ^**− 5**^		0.85 (0.73–1.03)	0.054
rs9268644	CC	484 (61.11)	631 (56.24)	998 (66.53)	Reference			Reference	
	CA	264 (33.33)	365 (32.53)	394 (26.27)	**0.72 (0.66–0.88)**	**0.001**		**0.68 (0.57–0.81)**	**1.32 × 10** ^**− 5**^
	AA	44 (5.56)	126 (11.23)	108 (7.20)	1.19 (0.83–1.72)	0.352		**0.54 (0.41–0.71)**	**1.26 × 10** ^**− 5**^
	CA + AA	308 (38.89)	491 (43.76)	502 (33.47)	**0.79 (0.66–0.95)**	**0.010**		**0.64 (0.55–0.76)**	**6.33 × 10** ^**− 8**^
rs35445101	AA	531 (67.05)	770 (68.63)	1152 (76.80)	Reference			Reference	
	AG	95 (11.99)	130 (11.59)	158 (10.53)	0.78 (0.59–1.02)	0.071		0.81 (0.63–1.04)	0.102
	GG	166 (20.96)	222 (19.79)	190 (12.67)	**0.53 (0.42–0.67)**	**7.38 × 10** ^**− 8**^		**0.57 (0.46–0.71)**	**3.18 × 10** ^**− 7**^
	AG + GG	261 (32.95)	352 (31.37)	348 (23.20)	**0.62 (0.51–0.75)**	**7.64 × 10** ^**− 7**^		**0.66 (0.56–0.79)**	**3.06 × 10** ^**− 6**^
rs24755213	AA	268 (33.84)	404 (36.01)	609 (40.60)	Reference			Reference	
	AG	392 (49.49)	508 (45.28)	635 (42.33)	**0.74 (0.61–0.89)**	**0.002**		0.85 (0.72–1.02)	0.073
	GG	132 (16.67)	210 (18.71)	256 (17.07)	0.88 (0.68–1.14)	0.323		0.83 (0.67–1.04)	0.104
	AG + GG	524 (66.16)	718 (63.99)	891 (59.40)	**0.77 (0.64–0.93)**	**0.005**		0.85 (0.72–0.99)	0.044
rs984778	TT	358 (45.20)	603 (53.74)	813 (54.20)	Reference			Reference	
	TC	344 (43.43)	429 (38.24)	574 (38.27)	**0.74 (0.61–0.88)**	**0.001**		1.02 (0.86–1.20)	0.858
	CC	90 (11.37)	90 (8.02)	113 (7.53)	**0.55 (0.41–0.74)**	**1.13 × 10** ^**− 4**^		0.94 (0.70–1.27)	0.693
	TC + CC	434 (54.80)	519 (46.26)	687 (45.80)	**0.70 (0.55–0.89)**	**0.004**		0.99 (0.85–1.17)	0.976

^a^ AOR, odds ratio adjusted for age, gender, ALT, AST in the comparison between HCC and Healthy controls

^b^ AOR, odds ratio adjusted for age, gender, HBeAg, HBV DNA load, ALT, and AST in the comparison between HCC and HBV-infected subjects without HCC.

*CI* confidence interval, *HBV* hepatitis B virus, *HCC* hepatocellular carcinoma, *HLA* human leukocyte antigen, *SNP* single nucleotide polymorphism

Boldface type indicates significant values, and the difference was significant. Statistically significant levels were corrected using the Bonferroni correction for multiple comparisons [*P* = 0.025 (two comparisons)]

### HCC-related HBV mutations

The EnhII/BCP/PC and preS regions were successfully amplified and sequenced from 1441 (72.52%) and 1045 (52.59%) of all genotypes B and C HBV-infected subjects, respectively. Higher age and more genotype B were observed in the HBV-infected subjects whose EnhII/BCP/PC regions were successfully sequenced than in those in whom this failed. Furthermore, there were significant differences in age, HBeAg positivity, and HBV DNA level between HBV-infected subjects whose preS regions were successfully sequenced than in those in whom this failed (Supplementary Table S3). No significant differences in the frequencies of rs3135363, rs9268644, rs35445101, rs24755213, and rs984778 were found between the HBV-infected subjects with successfully sequenced HBV regions and those not (Supplementary Table S4). The associations of hotspot mutations in the EnhII/BCP/PC and preS regions of HBV genome with HCC risk were shown in Table 3. In genotype B HBV-infected subjects, A1762T/G1764A, A1846T, and G1896A were significantly associated with an increased risk of HCC. Moreover, the frequencies of C1653T, T1674C/G, T1753A/C, A1762T/G1764A, G1719T, A1846T, G1896A, G1899A, and preS deletion increased successively along with the malignant transformation of chronic HBV infection in genotype C HBV-infected subjects.

**Table 3:** Associations of hotspot mutations related to HCC risks stratified by HBV genotypes

Hotspot mutation	CHB (%)	LC (%)	HCC (%)	HCC versus. HBV-infected subjects without HCC	
				AOR^a^ (95% CI)	*P* value
In genotype B HBV-infected subjects					
T1753A/C	6.12 (6/98)	15.00 (12/80)	8.14 (14/172)	0.57 (0.27–1.20)	0.139
A1762T/G1764A	34.69 (34/98)	37.50 (30/80)	60.47 (104/172)	**1.88 (1.19–2.98)**	**0.007**
A1846T	16.33 (16/98)	15.00 (12/80)	36.05 (62/172)	**2.00 (1.16–3.45)**	**0.013**
G1896A	16.33 (16/98)	20.00 (16/80)	43.02 (74/172)	**2.31 (1.36–3.93)**	**0.002**
In genotype C HBV-infected subjects					
C1653T	14.56 (46/316)	19.75 (64/324)	22.86 (198/866)	**1.55 (1.17–2.04)**	**0.002**
T1674C/G	15.82 (50/316)	14.81 (48/324)	24.25 (210/866)	**1.83 (1.38–2.44)**	**3.35 × 10** ^**− 5**^
T1753A/C	14.56 (46/316)	13.58 (44/324)	21.94 (190/866)	**1.89 (1.40–2.53)**	**2.47 × 10** ^**− 5**^
A1762T/G1764A	42.41 (134/316)	46.91 (152/324)	60.97 (528/866)	**2.09 (1.51–2.91)**	**1.13 × 10** ^**− 5**^
G1719T	36.08 (114/316)	39.51 (128/324)	44.57 (386/866)	**1.39 (1.06–1.83)**	**0.017**
A1846T	15.82 (50/316)	14.81 (48/324)	19.63 (170/866)	**1.53 (1.14–2.06)**	**0.005**
G1896A	27.85 (88/316)	15.43 (50/324)	30.25 (262/866)	**1.93 (1.47–2.54)**	**2.60 × 10** ^**− 6**^
G1899A	5.06 (16/316)	8.02 (26/324)	15.47 (134/866)	**2.96 (2.03–4.32)**	**1.77 × 10** ^**− 8**^
preS deletion	12.66 (40/316)	18.52 (60/324)	19.63 (170/866)	**1.77 (1.32–2.39)**	**1.53 × 10** ^**− 4**^

^a^ AOR, odds ratio adjusted for age, gender, HBeAg, HBV DNA load, ALT, and AST in the comparison between HCC and HBV-infected subjects with HCC.

*HBV* hepatitis B virus, *CHB* chronic hepatitis B, *LC* liver cirrhosis, *HCC* hepatocellular carcinoma

### Associations of ***HLA-DR*** genetic polymorphisms with HCC-related HBV mutations

We assessed the associations of *HLA-DR* genetic polymorphisms with the generation of HCC-related HBV mutations in genotypes B and C HBV-infected subjects, respectively, due to the different mutation patterns between HBV genotypes B and C (Table 4). In genotype C HBV-infected subjects, the CA genotype at rs9268644 was significantly associated with decreased frequencies of T1674C/G, A1762T/G1764A, G1899A, and preS deletion, and the significant associations of AA genotype with reduced frequencies of C1653T and T1753A/C were observed. There were significant associations of variant genotypes at rs35445101 with decreased frequencies of C1653T, A1762T/G1764A, G1719T, and G1896A. The variant genotypes at rs9268644 significantly decreased the generation of C1653T, A1762T/G1764A, G1719T, and G1896A, which were associated with the increased risks of HCC. The AG and GG genotypes at rs24755213 were associated with decreased frequencies of C1653T, T1674C/G, G1719T, T1753A/C A1762T/G1764A, A1846T, G1899A, and preS deletion. Decreased frequencies of C1653T, T1674C/G, G1719T, T1753A/C, A1762T/G1764A, A1846T, G1896A, and G1899A were observed in the subjects who were TC genotype at rs984778. The AG genotype at rs3135363, CA genotype at rs9268644, and AG genotype at rs24755213 were associated with decreased frequencies of T1753A/C, moreover, AG genotype at rs3135363 reduced the generation of G1896A in genotype B HBV-infected subjects.

**Table 4:** Significant associations of *HLA-DR* genetic polymorphisms with frequencies of HCC-related HBV mutations

SNPs	Genotype C HBV infected subjects				Genotype B HBV infected subjects		
	HCC-associated HBV mutation	AOR^a^ (95% CI)	*P* value		HCC-associated HBV mutation	AOR^a^ (95% CI)	*P* value
rs3135363							
AA		Reference				Reference	
AG					T1753A/C	0.33 (0.12–0.96)	0.042
					G1896A	0.20 (0.07–0.59)	0.003
rs9268644							
CC		Reference				Reference	
CA	T1674C/G	0.40 (0.24–0.68)	0.001		T1753A/C	0.34 (0.12–0.65)	0.033
	A1762T/G1764A	0.51 (0.28–0.92)	0.025				
	G1899A	0.43 (0.23–0.82)	0.010				
	preS deletion	0.55 (0.31–0.96)	0.036				
AA	C1653T	0.39 (0.16–0.95)	0.038				
	T1753A/C	0.33 (0.12–0.90)	0.030				
rs35445101							
AA		Reference				Reference	
AG	C1653T	0.33 (0.15–0.75)	0.008				
	A1762T/G1764A	0.37 (0.16–0.85)	0.019				
GG	C1653T	0.44 (0.21–0.93)	0.033				
	G1719T	0.37 (0.18–0.76)	0.007				
	G1896A	0.44 (0.21–0.89)	0.023				
rs24755213							
AA						Reference	
AG	C1653T	0.58 (0.38–0.88)	0.010		T1753A/C	0.26 (0.08–0.91)	0.035
	T1674C/G	0.45 (0.29–0.69)	3.23 × 10^− 4^				
	G1719T	0.65 (0.43–0.97)	0.035				
	A1762T/G1764A	0.50 (0.32–0.79)	0.003				
	A1846T	0.63 (0.39–0.99)	0.046				
	G1899A	0.36 (0.21–0.61)	1.32 × 10^− 4^				
	preS deletion	0.47 (0.29–0.75)	0.002				
GG	T1753A/C	0.33 (0.17–0.67)	0.002				
	A1846T	0.35 (0.16–0.75)	0.007				
	preS deletion	0.49 (0.25–0.97)	0.040				
rs984778							
TT							
TC	C1653T	0.37 (0.23–0.60)	4.15 × 10^− 5^				
	T1674C/G	0.34 (0.22–0.54)	4.00 × 10^− 6^				
	G1719T	0.46 (0.30–0.70)	3.01 × 10^− 4^				
	T1753A/C	0.54 (0.35–0.85)	0.007				
	A1762T/G1764A	0.61 (0.37–0.99)	0.046				
	A1846T	0.58 (0.36–0.92)	0.021				
	G1896A	0.51 (0.33–0.79)	0.002				
	G1899A	0.30 (0.16–0.55)	9.56 × 10^− 5^				

^a^ AOR, odds ratio adjusted for age, gender, HBeAg, HBV DNA load, ALT, and AST in the comparison between HCC and HBV-infected subjects without HCC.

*CI* confidence interval, *HBV* hepatitis B virus, *SNP* single nucleotide polymorphism

### Interaction between ***HLA-DR*** genetic polymorphisms and HBV mutations on the risk of HCC

In genotype C HBV-infected subjects, a significant association of T1753A/C or G1896A with decreased HCC risk was observed for those with AG genotype and G allele at rs3135363, respectively. The interactions of C1653T with CA genotype at rs9268644, and A1846T or G1896A with A allele at rs9268644 significantly reduced the risk of HCC. The interactions between AG genotype at rs24755213 with G1896A, G allele at rs24755213 with A1846T or G1896A were significantly associated with decreased risks of HCC (Supplementary Table S5). No significant interaction of HCC risk was observed in genotype B HBV-infected subjects.

## Discussion

It has been revealed that HBV infection can promote the development of HCC through multiple mechanisms. Among these, HBV infection-induced chronic inflammation leads to complex changes in the liver microenvironment. The interaction between HBV and innate and adaptive immune cells allows the virus to escape from immune surveillance and promotes disease progression from inflammation to fibrosis and eventually to HCC [6, 32]. Genetic predisposition, particularly variations in the HLA class II genes, has a significant impact on host immunity against HBV infection [10, 21, 33, 34]. Certain HLA class II alleles are associated with better control and clearance of HBV infection. For example, individuals with the variant alleles at rs3077(T), rs3135021(A), and rs9277535(A) located in the HLA-DP regions were associated with a lower risk of HBV persistence and a higher chance of spontaneous viral clearance [10]. However, some HLA class II alleles, such as rs2281388(T) allele at *HLA-DPB1* and rs9275319(A) allele at *HLA-DQ*, significantly increased the risk of chronic HBV infection and HCC [21, 35]. This study reported that variant alleles at rs3135363(G), rs9268644(A), rs35445101(G), rs24755213(G), and rs984778(C) were associated with decreased HCC risks in Han Chinese (Table 2). Genome-wide association studies (GWASs) had identified the associations of rs3135363 and rs984778 with differed antibody titer after HBV vaccination [28, 29]. The minor A allele of rs9268644 was associated with a reduced risk of hepatitis *via* enhancing the sensitivity of immune response [30]. Here, we firstly reported that the associations of variant G alleles at rs35445101 and rs24755213 with decreased HCC risks. Importantly, these alleles that predispose to decreased risks of HCC were less frequent in East Asian than in European populations [7]. This is likely one of reasons that HBV-infected HCC is endemic in East Asia.

Based on the definition of wild-type nucleotides of HBV genotypes B and C, we found that the frequencies of A1762T/G1764A, A1846T, and G1896A in HBV genotype B and C1653T, T1674C/G, T1753A/C, A1762T/G1764A, G1719T, A1846T, G1896A, G1899A, and preS deletion in HBV genotype C increased successively along with the malignant transformation of chronic HBV infection, which was quite consistent with previous findings [15, 16, 18, 36, 37]. HBV belongs to the hepadnaviridae, which is a family of enveloped viruses with an incomplete double-stranded DNA genome of 3.2 kb. According to the sequence divergence > 8% across the complete genome of HBV, HBV was classified into ten genotypes (A-J). Genotypes B and C were the predominant genotypes in East Asia [38, 39]. High rate of HBV replication and absence of proofreading activity in viral reverse transcriptase led to a mutation rate of 2.2 × 10^− 5^ substitutions/site/month, which was approximately 10-fold higher than other DNA virus [40–42]. During HBV-induced hepatocarcinogenesis, HBV experiences an evolutionary process characterized by the accumulation of HCC-related mutations such as C1653T, T1753A/C, and A1762T/G1764A [16, 18, 37]. Moreover, the mutations in HBV genome were usually concentrated in certain regions, particularly in the EnhII/BCP/PC and preS regions [15–18, 43, 44]. The frequencies of T1674C/G, C1653T, T1753A/C, and A1762T/G1764A were more than 30% in HBV-infected HCC patients [15]. A meta-analysis showed that PreS mutations, C1653T, T1753A/C, A1762T/G1764A were associated with 3.77-fold, 2.76-fold, 2.35-fold, and 3.79-fold increased risks of HCC compared to wild-type HBV, respectively [16]. Though HBV genotypes B and C had distinct mutation patterns, the mutations associated with increased HCC risk were similar between both genotypes (Table 3). We found that A1762T/G1764A, A1846T, and G1896A mutations were risk factors for HCC in genotypes B and C infections.

Chronic liver inflammation is often accompanied by the immune microenvironment which is involved a complex interplay between innate and adaptive immune cells as well as cytokines/chemokines in the occurrence of HCC [9]. The imbalance of Th1/Th2 cells, Treg/Th17 cells, neutrophil/lymphocyte, neutrophil/CD8^+^ T cell, Th1/Th2 cytokines, and disrupted inflammatory molecule networks might contribute to the non-resolving inflammation and promote the progression of HBV-related liver diseases [45]. The non-resolving inflammation is essential for immune selection of HCC-related HBV mutations [9, 46]. Genetic polymorphisms of HLA class II genes were engaged in the immune imbalance upon HBV infection, contributing to chronic HBV infections and non-resolving inflammation in liver [7, 22–24, 47]. For example, rs3077, rs9277535, and rs2281388 in HLA-DP region predisposed the host to suppress the expression of *HLA-DPA1* and *HLA-DPB1*, resulting in the downregulated Th1/Th2 ratio and subsequent HBV persistence [48]. In addition, rs477515-C might facilitate the Th1/Th2 transition *via* inhibiting the activity of *HLA-DRB1* enhancer, reduce the expression of *HLA-DRB1*, and prompt the immunoselection of HCC-related HBV mutations [7]. Our results suggested that genotype homozygous or heterozygous for the minor alleles of rs3135363, rs9268644, rs35445101, rs24755213, and rs984778 were associated with the decreased frequencies of HCC-related HBV mutations including A1762T/G1764A, C1653T, and T1753A/C (Table 4). Thus, it is speculated that *HLA-DR* genetic polymorphisms probably had an impact on the occurrence of HCC through modulating the immune selection of HBV mutations.

An important finding in this study was that *HLA-DR* genetic polymorphisms significantly affected the associations of HBV mutations with HCC risks in genotype C HBV-infected subjects (Supplementary Table S5). The tumor-promoting effects of C1653T, T1753A/C, A1846T, and G1896A mutations were significant only for those with genotype homozygous for the major alleles of rs3135363, rs9268644, and rs24755213 not for those with genotype heterozygous or the dominant model of minor alleles. The interactions of *HLA-DR* genetic polymorphisms with these HCC-related HBV mutations decreased the risks of HCC. Individuals with the interactions reducing HCC risks were less prevalent in the Han Chinese, implying that HBV-infected subjects of Han Chinese ethnicity were inherently susceptible to develop HCC. Further studies addressing the effects of these complicated interactions in hepatocarcinogenesis are urgently needed.

There were several limitations that should be acknowledged in this study. First, subjects enrolled in this study were not matched on the baseline characteristics including age, gender, HBeAg positivity, and HBV DNA load. Thus, we performed adjustment for these characteristics in the comparison among groups. Second, although study subjects included in this study were nongenetic relatives, rs35445101 did not conform to HWE. Third, the success rates of EnhII/BCP/PC and preS regions in HBV genome were less than 75% due to low HBV DNA load, leading to missing data on HBV mutations. Fourth, this study was designed as a case-control study, which had the inherent limitations.

## Conclusions

The present study revealed that variant genotypes of representative *HLA-DR* SNPs were associated with decreased risks of HCC, and these variant genotypes significantly decreased the generation of HCC-related HBV mutations. The interactions of these *HLA-DR* SNPs with HCC-related HBV mutations decreased the risks of HCC. *HLA-DR* genetic polymorphisms might predispose the host to immunoselection of HCC-related HBV mutations and affect the HCC risks *via* interacting with HBV mutations. This study may help in understanding the roles of *HLA-DR* genetic polymorphisms in the immune selection of HBV mutations and HCC development, which provides valuable insights into implementing active prophylaxis for HCC in HBV-infected subjects.

### Electronic supplementary material

Below is the link to the electronic supplementary material.

**Supplementary Material 1:** Supplementary methods HBV genotyping and HBV DNA sequencing and mutation analysis

**Supplementary Material 2: Supplementary Table S1** Probes and primers for genotyping of HLA-DR genetic polymorphisms, PCR reaction system, and condition

**Supplementary Material 3: Supplementary Table S2** Hardy-Weinberg tests in healthy controls

**Supplementary Material 4: Supplementary Table S3** Characteristics of HBV-infected subjects with/without successfully sequenced HBV regions

**Supplementary Material 5: Supplementary Table S4** Frequencies of *HLA-DR* genetic polymorphisms in HBV-infected subjects with/without successfully sequenced HBV regions

**Supplementary Material 6: Supplementary Table S5** Interactions of *HLA-DR* SNPs and HBV mutations on HCC risk in genotype C HBV-infected subjects

## Data Availability

The datasets used and analyzed are available from the corresponding author on reasonable request.
