# Supplementary material for: HLA-DR genetic polymorphisms and hepatitis B virus mutations affect the risk of hepatocellular carcinoma in Han Chinese population
Source: Virol J. 2023 Nov 30;20:283. doi: 10.1186/s12985-023-02253-2 (PMC10691135; doi:10.1186/s12985-023-02253-2)
Supplement: Supplementary file 1 — Supplementary Material 1: Supplementary methods HBV genotyping and HBV DNA sequencing and mutation analysis [file 12985_2023_2253_MOESM1_ESM.doc]

**Supplementary materials**

**HBV genotyping**

HBV genotypes were determined by a multiplex PCR assay that was developed in previous study [1]. Nested multiplex PCR was conducted for the genotyping of samples with low HBV DNA level. The primers for the first round of nest PCR are 5’-TTTGCGGGTCACCATATTCTTGG-3’ and 5’-CGAACCACTGAACAAATGG CACTAG-3’, which amplify a fragment of 1106bp from the preS/S region (nt.2815 to nt.705). The product of the first round PCR was used as the template for the multiplex PCR. The amplification conditions of the first round PCR and multiplex PCR were the same: 94°C for 5 min; 35 cycles which involves denaturing at 94°C for 30s, annealing at 58°C for 60s, and extending at 72°C for 1 min; a terminal extension step at 72°C for 5 min. The products were examined as previously examined [1].

**HBV DNA sequencing and mutation analysis**

The HBV enhancer II/basal core promoter/precore (EnhII/BCP/PC, nt.1636-nt.1900) and the preS (nt.2848-nt.155) regions were amplified by nested PCR. For EnhII/BCP/PC, the primers of the first round PCR were 5’-TGCACTTCGCTTC ACCTCTG-3’ and 5’-TAAGCGGGAGGAGTGCGAAT-3’, amplifying a fragment of 717bp (from nt.1594 to nt.2310); the primers of the second round PCR were 5’-TCGCATGGAGACCACCGTGA-3’ and 5’-ATAGCTTGCCTGAGTGC-3’, amplifying a fragment of 473bp (from nt.1604 to nt.2076). For preS region, the primers of the first round PCR were 5’-TGTGGAAGGCTGGCATT-3’ and 5’- AGAGGTTGGTGAGTGATTG-3’, amplifying a fragment of 803bp (from nt.2758 to nt.346); the primers of the second round PCR were 5’- TGGAAGGCTGGCATTCT-3’ and 5’- GGTATTGTGAGGATTCTTGTC-3’, amplifying a fragment of 695bp (from nt.2760 to nt.240). Sequence alignment and analysis were performed by using MEGA 4.0 software.

**References:**

1. Chen J, Yin J, Tan X, Zhang H, Zhang H, Chen B, et al. Improved multiplex-PCR to identify hepatitis B virus genotypes A-F and subgenotypes B1, B2, C1 and C2. J Clin Virol. 2007;38:238-43.
