# Supplementary material for: HLA-DR genetic polymorphisms and hepatitis B virus mutations affect the risk of hepatocellular carcinoma in Han Chinese population
Source: Virol J. 2023 Nov 30;20:283. doi: 10.1186/s12985-023-02253-2 (PMC10691135; doi:10.1186/s12985-023-02253-2)
Supplement: Supplementary file 2 — Supplementary Material 2: Supplementary Table S1 Probes and primers for genotyping of HLA-DR genetic polymorphisms, PCR reaction system, and condition [file 12985_2023_2253_MOESM2_ESM.docx]

**Supplementary Table S1** Probes and primers for genotyping of *HLA-DR* genetic polymorphisms, PCR reaction system, and condition

| SNPs | Probes and primers | Sequence (5' to 3') | PCR reaction system | PCR reaction condition |
| --- | --- | --- | --- | --- |
| rs3135363  (*HLA-DRA* -18kb A>G) | Probe 1 | FAM-AAGTAAGACGATTCTC-MGB | Reaction volume of 20 μL  containing 2.0 μL template DNA, 0.4 μL of each primer and probe (10 μmol/L), 50 U premix Taq (TaKaRa Biotechnology). | Step 1: 95ºC for 1 min  Step 2: 45 cycles of 95ºC for 10 sec and 60ºC for 30 sec  Step 3: 40ºC for 10 sec |
|  | Probe 2 | HEX-AAGTAAGGCGATTCTCA-MGB |  |  |
|  | Forward Primer | CGTGGCCATAGAGATCAAGGA |  |  |
|  | Reverse Primer | CAAAACGAAGACAGGAATAAAG |  |  |
| rs9268644  (*HLA-DRA* +380bp C>A) | Probe 1 | FAM-AATAGCACGGTCCTG-MGB |  |  |
|  | Probe 2 | HEX-AATAGCAAGGTCCTGC-MGB |  |  |
|  | Forward Primer | TTTAGAGGGTCAAAACTGAGTT |  |  |
|  | Reverse Primer | GAATATTTTCTGGCAAGCATTAAC |  |  |
| rs35445101  (*HLA-DRB1* +10.7kb A>G) | Probe 1 | FAM-CTCAGGAATCCTGCAA-MGB |  |  |
|  | Probe 2 | HEX-CTCAGGAGTCCTGCAAA-MGB |  |  |
|  | Forward Primer | GTTCTTCCTTGAATGTGGTCAT |  |  |
|  | Reverse Primer | AGTGTTGTTTTCAACCTGGCTCTA |  |  |
| rs24755213  (*HLA-DRB1* A>G) | Probe 1 | FAM-TTCCTACTGTCTGTCT-MGB |  |  |
|  | Probe 2 | HEX-TTCCTGCTGTCTGTCTG-MGB |  |  |
|  | Forward Primer | GTTTCTTGAAGCAGGATAAGTT |  |  |
|  | Reverse Primer | CCCGTAGTTGTGTCTGCACAC |  |  |
| rs984778  (*HLA-DRA* -7.5kb T>C) | Probe 1 | FAM-CCAAGGATATGTTTGT-MGB |  |  |
|  | Probe 2 | HEX-CCAAGGACATGTTTGTG-MGB |  |  |
|  | Forward Primer | AGTCACATATAAACATAGTATAC |  |  |
|  | Reverse Primer | TGTTTTGAGTGGACTGGTTCTTGGA |  |  |

*SNPs* single nucleotide polymorphisms, *FAM* 6-carboxyﬂuorescein, *HEX* 6-hexachloro-fluorescein, *MGB* minor groove binder, *HLA* human leukocyte antigen, *PCR* polymerase chain reaction.
