# Supplementary material for: HLA-DR genetic polymorphisms and hepatitis B virus mutations affect the risk of hepatocellular carcinoma in Han Chinese population
Source: Virol J. 2023 Nov 30;20:283. doi: 10.1186/s12985-023-02253-2 (PMC10691135; doi:10.1186/s12985-023-02253-2)
Supplement: Supplementary file 3 — Supplementary Material 3: Supplementary Table S2 Hardy-Weinberg tests in healthy controls [file 12985_2023_2253_MOESM3_ESM.docx]

**Supplementary Table S2** Hardy-Weinberg tests in healthy controls

| SNPs | Successful detection rate (%) | Genotype | n | *χ^2^* | *P* value |
| --- | --- | --- | --- | --- | --- |
| rs3135363 | 98.87 | AA | 465 | 1.330 | 0.249 |
|  |  | AG | 276 |  |  |
|  |  | GG | 51 |  |  |
| rs9268644 | 98.74 | CC | 484 | 1.010 | 0.315 |
|  |  | CA | 264 |  |  |
|  |  | AA | 44 |  |  |
| rs35445101 | 99.18 | AA | 531 | 383.006 | <0.001 |
|  |  | AG | 95 |  |  |
|  |  | GG | 166 |  |  |
| rs24755213 | 98.86 | AA | 268 | 0.316 | 0.574 |
|  |  | AG | 392 |  |  |
|  |  | GG | 132 |  |  |
| rs984778 | 99.08 | TT | 358 | 0.285 | 0.593 |
|  |  | TC | 344 |  |  |
|  |  | CC | 90 |  |  |

*SNPs* single nucleotide polymorphisms.
