# Supplementary material for: HLA-DR genetic polymorphisms and hepatitis B virus mutations affect the risk of hepatocellular carcinoma in Han Chinese population
Source: Virol J. 2023 Nov 30;20:283. doi: 10.1186/s12985-023-02253-2 (PMC10691135; doi:10.1186/s12985-023-02253-2)
Supplement: Supplementary file 4 — Supplementary Material 4: Supplementary Table S3 Characteristics of HBV-infected subjects with/without successfully sequenced HBV regions [file 12985_2023_2253_MOESM4_ESM.docx]

**Supplementary Table S3** Characteristics of HBV-infected subjects with/without successfully sequenced HBV regions

| Characteristics | Sequencing of EnhII/BCP/PC region | | |  | Sequencing of preS region | | |
| --- | --- | --- | --- | --- | --- | --- | --- |
|  | Success (n = 1441) | Failure (n = 546) | *P* value |  | Success (n = 1045) | Failure (n = 942) | *P* value |
| Male (%) | 1132 (78.56) | 427 (78.21) | 0.865 |  | 813 (77.80) | 746 (79.19) | 0.450 |
| Age (mean ± SD) | 50.60 ± 12.32 | 49.33 ± 11.87 | 0.037 |  | 49.48 ± 12.61 | 51.10 ± 11.70 | 0.003 |
| HBV genotype (%) |  |  |  |  |  |  |  |
| B | 335 (23.25) | 81 (7.14) | < 0.001 |  | 186 (17.80) | 188 (19.96) | 0.219 |
| C | 1169 (76.75) | 373 (92.86) |  |  | 859 (82.20) | 754 (80.04) |  |
| HBeAg (%) |  |  |  |  |  |  |  |
| Positive | 520 (37.49) | 172 (32.70) | 0.051 |  | 445 (44.28) | 247 (27.20) | < 0.001 |
| Negative | 867 (62.51) | 354 (67.30) |  |  | 560 (55.72) | 661 (72.80) |  |
| HBV DNA (log_10_ IU/mL) | 3.65 ± 0.65 | 3.55 ± 0.70 | 0.102 |  | 3.86 ± 0.68 | 3.34 ± 0.59 | < 0.001 |

The information of genotype B and C HBV-infected subjects (n=1987) were listed.

*EnhII/BCP/PC* enhancer II/basal core promoter/precure, *HBV* hepatitis B virus, *HBeAg* hepatitis B e antigen, *SD* Standard deviation.
