# Supplementary material for: HLA-DR genetic polymorphisms and hepatitis B virus mutations affect the risk of hepatocellular carcinoma in Han Chinese population
Source: Virol J. 2023 Nov 30;20:283. doi: 10.1186/s12985-023-02253-2 (PMC10691135; doi:10.1186/s12985-023-02253-2)
Supplement: Supplementary file 5 — Supplementary Material 5: Supplementary Table S4 Frequencies of HLA-DR genetic polymorphisms in HBV-infected subjects with/without successfully sequenced HBV regions [file 12985_2023_2253_MOESM5_ESM.docx]

**Supplementary Table S4** Frequencies of *HLA-DR* genetic polymorphisms in HBV-infected subjects with/without successfully sequenced HBV regions

| SNPs | Genotype | HBV-infected subjects with successfully sequenced HBV regions (n = 1181) | HBV-infected subjects without successfully sequenced HBV regions (n = 806) | *P* value |
| --- | --- | --- | --- | --- |
| rs3135363 | AA | 773 (65.45%) | 533 (66.13%) | 0.246 |
|  | AG | 351 (29.72%) | 222 (27.54%) |  |
|  | GG | 57 (4.83%) | 51 (6.33%) |  |
| rs9268644 | CC | 708 (59.95%) | 510 (63.28%) | 0.218 |
|  | CA | 359 (30.40%) | 216 (26.80%) |  |
|  | AA | 114 (9.65%) | 80 (9.92%) |  |
| rs35445101 | AA | 875 (74.09%) | 562 (69.73%) | 0.075 |
|  | AG | 129 (10.92%) | 111 (13.77%) |  |
|  | GG | 177 (14.99%) | 133 (16.50%) |  |
| rs24755213 | AA | 430 (36.41%) | 327 (40.57%) | 0.165 |
|  | AG | 525 (44.45%) | 331 (41.07%) |  |
|  | GG | 226 (19.14%) | 148 (18.36%) |  |
| rs984778 | TT | 635 (53.77%) | 443 (54.96%) | 0.864 |
|  | TC | 453 (38.36%) | 300 (37.22%) |  |
|  | CC | 93 (7.87%) | 63 (7.82%) |  |

*HBV* hepatitis B virus, *SNPs* single nucleotide polymorphisms.
