# Supplementary material for: HLA-DR genetic polymorphisms and hepatitis B virus mutations affect the risk of hepatocellular carcinoma in Han Chinese population
Source: Virol J. 2023 Nov 30;20:283. doi: 10.1186/s12985-023-02253-2 (PMC10691135; doi:10.1186/s12985-023-02253-2)
Supplement: Supplementary file 6 — Supplementary Material 6: Supplementary Table S5 Interactions of HLA-DR SNPs and HBV mutations on HCC risk in genotype C HBV-infected subjects [file 12985_2023_2253_MOESM6_ESM.docx]

**Supplementary Table S5** Interactions of *HLA-DR* SNPs and HBV mutations on HCC risk in genotype C HBV-infected subjects

| SNPs | HBV mutations | CHB plus LC | HCC | AOR^a^ (95% CI) | *P* value |
| --- | --- | --- | --- | --- | --- |
| rs3135363 | T1753A/C |  |  |  |  |
| AA | T | 210 | 226 | Reference |  |
| AA | A/C | 48 | 132 | **2.56 (1.75-3.74)** | **1.31×10^-6^** |
| AG | T | 78 | 100 | 1.19 (0.84-1.69) | 0.328 |
| AG | A/C | 33 | 50 | **0.55 (0.32-0.96)** | **0.034** |
| For interaction |  |  |  | **0.46 (0.24-0.89)** | **0.020** |
| rs3135363 | G1896A |  |  |  |  |
| AA | G | 166 | 156 | Reference |  |
| AA | A | 82 | 186 | **2.41 (1.72-3.39)** | **3.64×10^-7^** |
| AG | G | 54 | 66 | 1.30 (0.85-1.98) | 0.221 |
| AG | A | 46 | 66 | **0.63 (0.40-0.99)** | **0.049** |
| For interaction |  |  |  | **0.49 (0.26-0.91)** | **0.023** |
| rs3135363 | T1753A/C |  |  |  |  |
| AA | T | 210 | 226 | Reference |  |
| AA | A/C | 48 | 132 | **2.56 (1.75-3.74)** | **1.31×10^-6^** |
| AG+GG | T | 92 | 112 | 1.13 (0.81-1.58) | 0.469 |
| AG+GG | A/C | 43 | 58 | **0.49 (0.29-0.82)** | **0.007** |
| For interaction |  |  |  | **0.43 (0.24-0.80)** | **0.008** |
| rs3135363 | G1896A |  |  |  |  |
| AA | G | 166 | 156 | Reference |  |
| AA | A | 82 | 186 | **2.41 (1.72-3.39)** | **3.64×10^-7^** |
| AG+GG | G | 70 | 76 | 1.16 (0.78-1.71) | 0.470 |
| AG+GG | A | 56 | 76 | **0.60 (0.39-0.92)** | **0.020** |
| For interaction |  |  |  | **0.52 (0.29-0.93)** | **0.027** |
| rs9268644 | C1653T |  |  |  |  |
| CC | C | 178 | 244 | Reference |  |
| CC | T | 56 | 140 | **1.82 (1.27-2.63)** | **0.001** |
| CA | C | 88 | 88 | **0.57 (0.40-0.80)** | **0.001** |
| CA | T | 42 | 40 | 0.62 (0.36-1.09) | 0.094 |
| For interaction |  |  |  | **0.52 (0.28-0.99)** | **0.046** |
| rs9268644 | A1846T |  |  |  |  |
| CC | A | 154 | 210 | Reference |  |
| CC | T | 48 | 128 | **1.96 (1.32-2.89)** | **0.001** |
| CA+AA | A | 116 | 96 | **0.61 (0.43-0.85)** | **0.004** |
| CA+AA | T | 50 | 42 | **0.32 (0.19-0.53)** | **1.77×10^-5^** |
| For interaction |  |  |  | **0.52 (0.28-0.97)** | **0.041** |
| rs9268644 | G1896A |  |  |  |  |
| CC | G | 140 | 160 | Reference |  |
| CC | A | 64 | 186 | **2.54 (1.77-3.66)** | **4.84×10^-7^** |
| CA+AA | G | 96 | 72 | **0.66 (0.45-0.96)** | **0.030** |
| CA+AA | A | 74 | 76 | **0.35 (0.23-0.54)** | **1.90×10^-6^** |
| For interaction |  |  |  | **0.54 (0.30-0.96)** | **0.034** |
| rs24755213 | G1896A |  |  |  |  |
| AA | G | 86 | 84 | Reference |  |
| AA | A | 30 | 128 | **4.37 (2.65-7.19)** | **6.70×10^-9^** |
| AG | G | 108 | 102 | 0.97 (0.65-1.45) | 0.871 |
| AG | A | 64 | 90 | **0.33 (0.20-0.55)** | **2.04×10^-5^** |
| For interaction |  |  |  | **0.34 (0.18-0.65)** | **0.001** |
| rs24755213 | A1846T |  |  |  |  |
| AA | A | 88 | 118 | Reference |  |
| AA | T | 26 | 88 | **2.52 (1.51-4.23)** | **4.52×10^-4^** |
| AG+GG | A | 176 | 182 | 0.77 (0.55-1.09) | 0.140 |
| AG+GG | T | 70 | 76 | **0.32 (0.19-0.55)** | **4.31×10^-5^** |
| For interaction |  |  |  | **0.42 (0.22-0.79)** | **0.008** |
| rs24755213 | G1896A |  |  |  |  |
| AA | G | 86 | 84 | Reference |  |
| AA | A | 30 | 128 | **4.37 (2.65-7.19)** | **6.70×10^-9^** |
| AG+GG | G | 146 | 144 | 1.01 (0.69-1.48) | 0.960 |
| AG+GG | A | 104 | 126 | **0.28 (0.18-0.46)** | **2.03×10^-7^** |
| For interaction |  |  |  | **0.28 (0.15-0.52)** | **4.24×10^-5^** |

^a^AOR odds ratio adjusted for age, gender and serum HBV DNA load.

*CI* confidence interval, *HBV* hepatitis B virus, *HCC* hepatocellular carcinoma, *HLA* human leukocyte antigen, *SNPs* single nucleotide polymorphisms.
